# A Case of Primary Pulmonary Meningioma With Genetic Characterization and Literature Review

**DOI:** 10.1155/crom/6077936

**Published:** 2025-09-30

**Authors:** Zachary Braunstein, Jesse Pullen, Konstantin Shilo, Andres Madrigal, Gabriel Tinoco

**Affiliations:** ^1^Department of Internal Medicine, Division of Medical Oncology, The Ohio State University Comprehensive Cancer Center, Columbus, Ohio, USA; ^2^Department of Pathology, The Ohio State University Comprehensive Cancer Center, Columbus, Ohio, USA

## Abstract

Primary pulmonary meningiomas (PPMs) are exceptionally rare tumors, accounting for a small fraction of ectopic meningiomas found outside the central nervous system (CNS). These tumors are typically benign, with only 5%–8% being malignant. Since the first reported case in 1982, fewer than 100 cases have been documented. We report a unique case of PPM in a 58-year-old male detected incidentally during lung cancer screening. Histopathological and immunohistochemical (IHC) analysis confirmed the diagnosis, and next-generation sequencing (NGS) revealed a Neurofibromatosis Type 2 (NF2) inactivating mutation and a likely subclonal PALB2 mutation—findings not commonly reported in the PPM literature. Our comprehensive review of 70 reported cases reveals a female predominance, asymptomatic presentation in most cases, and strong IHC positivity for EMA, PR, and vimentin. Malignant cases tended to be larger and more symptomatic. Our case highlights the value of molecular profiling in differentiating PPMs from other pulmonary neoplasms and CNS metastases. Given the emerging role of NGS in identifying tumor-specific mutations, further exploration of the genetic landscape of PPMs is warranted. Recognition of NF2 mutations as potential drivers may open new avenues for targeted therapies and long-term monitoring strategies in affected individuals.

## 1. Introduction

Meningiomas are one of the most common primary central nervous system (CNS) tumors, accounting for over one-third of CNS tumors and half of all benign brain tumors [1]. Primary CNS meningiomas are typically benign, with a very low likelihood of metastases, although 1%–3% can be malignant [2]. Rarely, ectopic meningiomas (1%–2% of meningiomas) can occur in locations including the head and neck, peripheral nervous system (PNS), skin, and thoracic cavity [3, 4]. Since the first case report describing primary pulmonary meningiomas (PPMs) in 1982 [5], less than 70 cases have been described in the literature [6]; none have described somatic mutations associated with the diagnosis. Here, we discuss a case of PPM with associated somatic mutations and review the available literature.

## 2. Case Presentation

**Figure 1:**
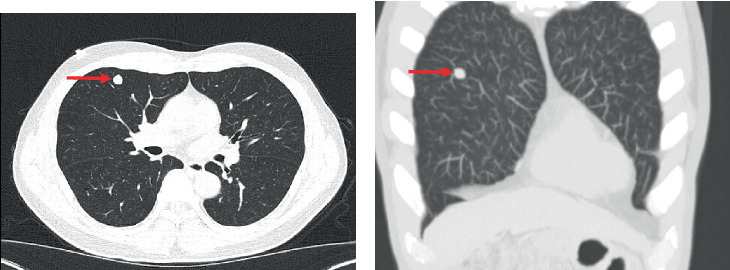
Low-dose noncontrast CT (lung cancer screening protocol) showing a 1-cm right upper lobe pulmonary nodule in both (a) axial view and (b) coronal view.

A 58-year-old male with a past medical history of hypertension, hyperlipidemia, chronic hepatitis B, and a 15-pack-year smoking history underwent lung cancer screening via chest computed tomography (CT), which identified a 1.0 × 1.0-cm noncalcified nodule in the right upper lobe (RUL) (Figure 1).

**Figure 2:**
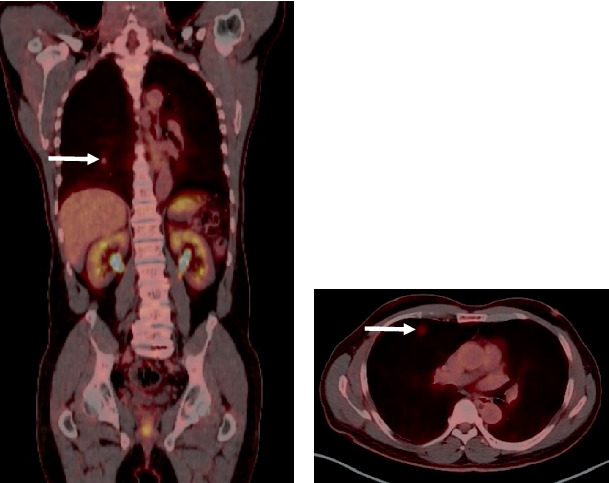
FDG positron emission tomography (PET) scan showing a 1-cm right upper lobe pulmonary nodule with mild FDG avidity (SUV 2.1) in both (a) coronal view and (b) axial view.

Retrospective analysis of prior imaging revealed interval growth from 0.6 × 0.6 cm over 2.5 years. Subsequent positron emission tomography (PET)–CT demonstrated mild metabolic activity with a maximum standardized uptake value (SUV) of 2.1 (mediastinal SUV: 1.6; hepatic SUV: 2.8) (Figure 2), with no additional hypermetabolic lesions identified.

**Figure 3:**
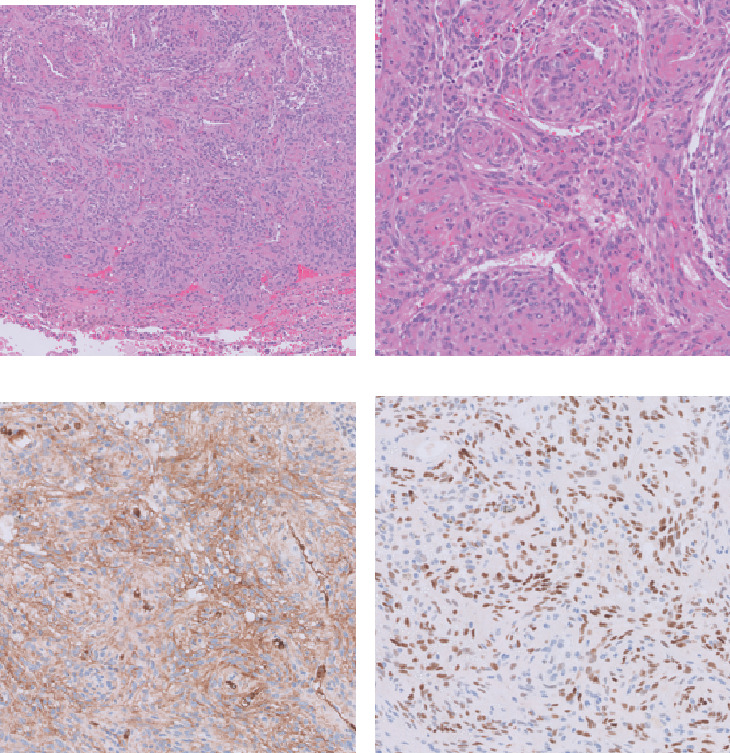
Pathological findings. The tumor represents a (a) small, well-circumscribed mass that is composed of spindle-to-epithelioid cells that form (b) characteristic whorls; H&E, original magnification ×5 and ×20, respectively. The tumor shows diffuse expression of (c) epithelial membrane antigen and (d) progesterone receptors; immunohistochemistry, original magnification ×10.

Navigational bronchoscopy with biopsy identified a low-grade spindle-cell neoplasm and revealed *Aspergillus* colonization; however, infectious disease consultation recommended no antifungal treatment due to the absence of clinical infection. The patient underwent RUL wedge resection. Gross examination revealed a 1.1 × 0.9 × 0.7-cm friable, well-circumscribed lesion with 0.6-cm margins. Histopathological analysis demonstrated a well-demarcated mass composed of spindle-to-epithelioid cells arranged in characteristic whorled patterns (Figure 3a,b). The tumor cells were arranged in solid nests, lobules, and papillary structures. The tumor cells had a minimal to moderate amount of eosinophilic cytoplasm and bland round to oval nuclei, some containing intranuclear inclusions. While the tumor had areas of increased cellularity, there was no necrosis and only rare mitosis (less than 1 per 2 mm^2^) with a Ki-67 proliferation index not exceeding 1%.

Immunohistochemistry (IHC) staining showed positivity for epithelial membrane antigen (EMA) (Figure 3c), progesterone receptor (PR) (Figure 3d), and smooth muscle antigen (SMA) and showed negativity for SRY-box transcription factor 10 (SOX10), desmin, cluster of differentiation 34 (CD34), cytokeratin AE 1/AE 3, and thyroid transcription factor 1 (TTF-1).

Next-generation sequencing (NGS) was performed using Tempus xT, a targeted panel sequencing assay that evaluates 648 cancer-associated genes for single-nucleotide variants, insertions, deletions, copy number variations, and structural rearrangements. DNA extracted from formalin-fixed paraffin-embedded (FFPE) tissue was sequenced at an average depth of > 500X.

Tempus xT targeted NGS identified a Neurofibromatosis Type 2 (NF2) frameshift mutation (NF2 c.1633_1634del, p.E545fs) with a variant allele frequency (VAF) of 44.7% and a subclonal PALB2 frameshift mutation (PALB2 c.1570del, p.S524fs) with a VAF of 9.7%. No mutations were detected in other common meningioma-associated genes, including AKT1, TRAF7, KLF4, or SMO. A neurotrophic tyrosine receptor kinase (NTRK) fusion panel was negative. The pathological and IHC findings were consistent with a meningioma. Brain magnetic resonance imaging (MRI) revealed no abnormalities, supporting the PPM diagnosis. Five months following surgical resection, the patient has shown no evidence of recurrence; repeat PET scan showed no hypermetabolic activity.

## 3. Discussion

CNS meningiomas are the most common primary intracranial tumor and are generally considered a benign disease [7]. Histologic features of meningiomas are typically a spherical formation of meningothelial cells, called whorls, which eventually mineralize into psammoma bodies. It is also common to see central chromatin clearing and intranuclear cytoplasmic pseudoinclusions [7]. Typical IHC findings for CNS meningiomas are EMA, somatostatin receptor 2A (SST2A), PR (~70%–80% of cases), and vimentin [8, 9]. The most common familial syndrome associated with meningiomas is NF2, although this is seen in < 1% of meningioma cases [7, 10]. Malignant meningiomas (World Health Organization [WHO] Grade III) represent ~1% of all meningiomas; they can arise either as progression of benign meningiomas or de novo [11]. The main treatment for malignant meningioma is surgical resection with postoperative radiation [8, 12].

PPMs are an uncommon condition with less than 70 reported cases in the literature. The exact physiopathology of this condition has not been fully elucidated. Still, it is thought that they either arise from minute pulmonary meningothelial-like nodules [13] or migrate with developing nerves or blood vessels as they pass through cranial openings [14]. A literature review, including our case, identified 70 reported cases of PPM, with a mean diagnostic age of 56 years (range: 18–81) and a predominance in females (64%). The majority (67%, *n* = 47) were asymptomatic at the time of diagnosis. The most common tumor location was the left upper lobe (LUL) (27%, *n* = 19), followed by the RUL (26%, *n* = 18). Coexisting malignancies were present in 16% (*n* = 11) of cases. IHC analysis most frequently demonstrated positivity for EMA (98%, 59/60), vimentin (96%, 43/45), and PR (94%, 30/32). A full breakdown of baseline characteristics can be seen in Table 1 and Table S1.

Pathological diagnosis is necessary for a PPM diagnosis due to the nonconformity of how the lesions appear on imaging. While almost three-quarters of PPMs are less than 4 cm [6], some as large as 15 cm have been reported (mean size of 3 cm [range: 0.5–15 cm]). The histopathology of PPM overlaps with several pulmonary spindle cell lesions. These include benign to low-grade malignant tumors (schwannoma, leiomyoma, inflammatory myofibroblastic tumor [IMT], solitary fibrous tumor [SFT], synovial sarcoma [SS], and recently described low-grade fibromyxoid sarcoma [LGFMS]). These can be distinguished from each other with some certainty based on light microscopic features and IHC. While meningiomas typically express vimentin, EMA, and PR, they lack expression of S100 or SOX10, as observed in schwannoma [15], or expression of ALK1, as seen in IMT [16]. Meningiomas do not express CD34 or STAT6, typically seen in SFT [17], and they lack expression of MUC4, as seen in LGFMS. Furthermore, the presence of characteristic genetic alterations in IMT, SFT, LGFMS, and SS, determined through molecular testing, is helpful in distinguishing these tumors from PPM.

Our review of 70 individual PPM cases (Table S1) shows that the majority were asymptomatic at diagnosis, with lesions discovered incidentally. The most frequent tumor locations were the upper lobes, and there was a slight female predominance. While most tumors were under 4 cm in size and benign, malignant cases tended to be larger and associated with symptoms such as cough or chest pain. IHC staining was highly consistent across cases, with near-universal positivity for EMA, PR, and vimentin. These observations help refine the clinical picture of PPMs and highlight distinguishing features that may aid in identifying malignant variants.

When breaking down the cases between benign (*n* = 65) and malignant (*n* = 5), the frequent nature of being positive for vimentin (95% in benign; 100% in malignant), PR (93% in benign; 100% in malignant), and EMA (98% in benign; 100% in malignant) was consistent among cases where it was evaluated for. While caution must be taken with small numbers, the mean size at diagnosis was larger for malignant cases at 6.2 cm compared to 2.8 cm for benign cases. The frequency of presenting with symptoms was increased in malignant patients, as only 40% were asymptomatic; 71% of benign patients presented asymptomatically.

Our case is unique in that we have an NGS-based gene panel report from resected meningioma tissue. Our patient has an *NF2* inactivating mutation (E545fs) with a VAF of 44.7% and a likely subclonal PALB2 mutation with a VAF of 9.7%. NF2 disease is characterized by both CNS and PNS tumors, with the most common being meningiomas [18]. A limitation of our report is the absence of cytogenetic or chromosomal microarray analyses. Thus, we did not evaluate common meningioma-associated cytogenetic abnormalities (e.g., chromosome 22q deletion or alterations in chromosomes 1, 6, 10, 14, 18, and 19).

NGS of PPMs has found genetic changes similar to those in brain meningiomas. Common mutations occur in genes including NF2, TRAF7, KLF4, AKT1, and SMO, which are linked to tumor growth [19–22]. PPMs usually have a low number of mutations and a stable genome, matching their slow-growing nature [23]. Unlike primary lung cancers, PPMs do not have mutations in EGFR, KRAS, ALK, and ROS1, highlighting their unique development.

In contrast, molecular characterization of meningiomas typically identifies mutations in NF2, TRAF7, AKT1, SMO, KLF4, POLR2A, TERT, and PIK3CA, as well as recurrent chromosomal abnormalities involving 22q loss, 1p deletion, and alterations in 6q, 14q, and 18q [24]. Bukovac et al. [25] examined nucleotide variations in TP53 Exon 4 in intracranial meningiomas and employed in silico modeling to evaluate their possible functional impacts. This highlights the importance of expanding molecular profiling to include TP53 and associated pathways in future meningioma research. Overall, these findings provide important context for interpreting the molecular profile observed in our case. *NF2* mutations have been specifically evaluated for previously in PPM, with one case being identified [26] and a pediatric case of NF2 showing recurrent pulmonary meningiomas [27].

Our case underscores the critical role of molecular profiling in differentiating PPMs from primary lung malignancies, ensuring precise diagnosis and optimal management. Given the rarity of PPMs, NGS is pivotal in identifying key genetic alterations that inform prognosis and therapeutic strategies. Elucidating the molecular landscape of PPMs not only aids in distinguishing them from metastatic meningiomas but also enhances our understanding of their pathogenesis.

The NF2 gene encodes the tumor suppressor protein merlin, which regulates cell proliferation and cell–cell interactions; its inactivation is a known driver in CNS meningiomas [28]. Partner and localizer of BRCA2 (PALB2) interacts with BRCA1/2 in homologous recombination repair, serving as a tumor suppressor. Mutations in PALB2 have been widely studied in breast and ovarian cancers, though not previously associated with meningiomas [29].

This case shows that *NF2* mutation could potentially be a driving factor for the development of meningiomas outside of the CNS. Even though our patient had benign pathology, follow-up imaging should be considered to verify that the patient does not have recurrent meningiomas deriving from his biallelic NF2 mutation. Further research is warranted to explore targeted therapies based on identified genetic alterations, particularly in cases exhibiting atypical or aggressive features.

## 4. Conclusion

This case adds to the small but growing body of literature on PPMs, underscoring the value of molecular profiling in diagnosis and clinical decision-making. The patient's NGS results revealed an NF2 inactivating mutation and a likely subclonal PALB2 mutation—alterations not commonly reported in this setting.

NF2 mutations are well established in CNS meningiomas and may also play a key role in the pathogenesis of ectopic tumors like PPMs. The PALB2 finding is intriguing but remains of uncertain relevance in this context. The novelty of this case comprises a comprehensive characterization of PPM utilizing NGS testing that confirms its genetic similarity to a CNS counterpart. The presence of a specific molecular alteration (NF2 mutation) in PPM can serve as a helpful tool in separating PPM from other spindle cell lesions in the lungs. Our literature review supports the knowledge that PPMs are typically benign, slow-growing, and asymptomatic, with a consistent IHC profile including EMA, PR, and vimentin. Malignant variants, though rare, tend to present at larger sizes and with more symptoms.

This case also highlights the diagnostic complexity these lesions can present, particularly when trying to distinguish them from primary lung malignancies or metastatic disease. In such scenarios, genomic data can offer essential clarity. Given their rarity, routine molecular testing may help guide diagnosis, follow-up, and future therapeutic strategies. As more cases are molecularly characterized, we will be better positioned to understand the biology of PPMs and refine our clinical approach accordingly.

## Figures and Tables

**Table 1:** Baseline combined characteristics.

**Characteristic**	**All patients (** **n** = 70**)**	**Benign histology (** **n** = 65**)**	**Malignant histology (** **n** = 5**)**
Mean age at diagnosis (range)	56 (18–81)	56 (19–79)	56 (40–81)
Female sex	63% (44/70)	63% (41/65)	60% (3/5)
Additional malignancy	16% (11/70)	15% (10/65)	20% (1/5)
Mean SUV on PET scan (range)	4.88 (0.6–12.9) (*n* = 12)	4.90 (0.6–12.9) (*n* = 11)	4.63 (*n* = 1)
Vimentin IHC positive	96% (43/45)	95% (38/40)	100% (5/5)
EMA IHC positive	98% (59/60)	98% (54/55)	100% (5/5)
Progesterone receptor IHC positive	94% (30/32)	93% (27/29)	100% (3/3)
S100 or SOX10 IHC positive	34% (12/35)	33% (11/33)	50% (1/2)
Cytokeratin A/E positive	9% (2/23)	9% (2/22)	0% (0/1)
Desmin positive	0% (0/22)	0% (0/22)	Unk
CD34 positive	24% (6/25)	22% (5/23)	50% (1/2)
Location			
LUL	27% (19/70)	28% (18/65)	20% (1/5)
LLL	14% (10/70)	14% (9/65)	20% (1/5)
RUL	11% (8/70)	11% (7/65)	20% (1/5)
RML	4% (4/70)	5% (3/65)	0% (0/5)
RLL	26% (18/70)	26% (17/65)	20% (1/5)
Multiple sites	10% (7/70)	9% (6/65)	20% (1/5)
Unknown	7% (5/70)	8% (5/65)	0% (0/5)
Mean size at diagnosis (cm) (range)	3.0 (0.5–15.0)	2.8 (0.5–15.0)	6.2 (1.2–12.0)
Symptoms at diagnosis^a^			
Asymptomatic	69% (48/70)	71% (46/65)	40% (2/5)
Bland cough	13% (9/70)	11% (7/65)	40% (2/5)
Hemoptysis	4% (3/70)	5% (3/65)	0% (0/5)
Chest pain	9% (6/70)	8% (5/65)	20% (1/5)
Shortness of breath	6% (4/70)	5% (3/65)	20% (1/5)
Fatigue	1% (1/70)	2% (1/65)	0% (0/5)
Pain from metastasis	1% (1/70)	0% (0/65)	20% (1/5)
Unknown	3% (2/70)	3% (2/65)	0% (0/5)

Abbreviations: CD, cluster of differentiation; EMA, epithelial membrane antigen; LLL, left lower lobe; LUL, left upper lobe; PET, positron emission tomography; PR, progesterone receptor; RLL, right lower lobe; RML, right middle lobe; RUL, right upper lobe; SOX, Sry-related HMG-box; Unk, unknown.

^a^Patients may have presented with more than one symptom.

## Data Availability

The data that support the findings of this study are available from the corresponding author upon reasonable request.
