# Supplementary material for: A Case of Primary Pulmonary Meningioma With Genetic Characterization and Literature Review
Source: Case Rep Oncol Med. 2025 Sep 30;2025:6077936. doi: 10.1155/crom/6077936 (PMC12503964; doi:10.1155/crom/6077936)
Supplement: Supporting Information — Additional supporting information can be found online in the Supporting Information section. Table S1: Breakdown of individual cases. [file 6077936.f1.docx]

**Table S1: Breakdown of Individual Cases**

| Case Report | Age (sex) | | Symptoms at Diagnosis | Location | Size (cm) (largest if multiple) | Additional Malignancy | SUV on PET Scan | EMA | PR | Vimentin | CD34 | Ki-67 | S100 or SOX10 | Histology | Treatment |
| --- | --- | --- | --- | --- | --- | --- | --- | --- | --- | --- | --- | --- | --- | --- | --- |
| Braunstein et al | Male (58) | None | | RUL | 1.0 | None | 2.1 | Positive | Positive | Unk | Negative | 1% | Negative | Benign | RUL Wedge Resection |
| Zhang et al^1^ | Female (47) | None | | LLL | 6.9 | None | 4.4 | Positive | Positive | Unk | Positive | 5% | Negative | Benign | open cuff resection of the LLL and wedge resection of the lingual segment |
| Dai et al^2^ | Female (59) | none | | RLL | 1.1 | None | Unk | Positive | Positive | Unk | Negative | 2% | Negative | Benign | Unk |
| Cesario et al^3^ | Male (56) | None | | LUL | 2.0 | None | Unk | Positive | Negative | Positive | Unk | Unk | Negative | Benign | LUL Wedge Resection |
| Yang et al^4^ | Male (41) | Chest Pain, Cough | | RUL, RLL | 12.0 | None | Unk | Positive | Unk | Positive | Positive | Unk | Unk | Malignant | RML And RUL lobectomies |
| Oh et al^5^ | Male (54) | None | | Scattered | 9.6 | None | 3.1 | Positive | Unk | Unk | Negative | Unk | Unk | Benign | Wedge Resection |
| Bas et al^6^ | Male (57) | Cough | | LLL | 1.1 | None | Unk | Positive | Positive | Positive | Negative | No mitosis observed | Unk | Benign | LLL wedge resection |
| Jiang et al^7^ | Male (70) | None | | RLL | 1.5 | Rectal Carcinoma | 0.6 | Positive | Unk | Positive | Unk | <5% | Unk | Benign | RLL Wedge resection |
| Gurcay et al^8^ | Female (55) | cough | | RUL | 2.0 | Uterine leomyoma | 1.89 | Positive | Positive | Positive | Unk | No mitosis observed | Positive | Benign | RUL Wedge resection |
| Fujikawa et al^9^ | Female (62) | None | | LLL | 0.9 | papillary thyroid carcinoma | Unk | Positive | Positive | Positive | Unk | Unk | Unk | Benign | LLL Wedge resection |
| Wang et al^10^ | Female (64) | None | | Scattered | 3.4 | None | Unk | Positive | Positive | Unk | Positive | 2% | Unk | Benign | Wedge resection |
| Han et al^11^ | Female (75) | None | | RLL | 0.6 | None | Unk | Positive | Positive | Positive | Negative | 1% | Negative | Benign | RLL Wedge resection |
| Han et al^11^ | Female (64) | None | | RUL, RLL | 0.5 | None | Unk | Positive | Positive | Positive | Unk | 1% | Negative | Benign | Wedge resection |
| Cimini et al.^12^ | Male (81) | None | | LUL | 1.2 | Thymoma; RCC | 4.63 | Positive | Positive | Positive | Unk | 20% | Unk | Malignant | LUL Wedge resection |
| Bae et al^13^ | Female (43) | None | | LUL | 1.9 | None | "increased" | Positive | Unk | Positive | Unk | Unk | Unk | Benign | LUL Wedge Resection |
| Ohashi-Nakatani et al^14^ | Female (60) | None | | RLL | 2.0 | None | Unk | Positive | Positive | Unk | Unk | Unk | Unk | Benign | RLL segmentectomy |
| Xu et al^15^ | Female (65) | Chest pain and tightness | | RLL | 0.7 | None | Unk | Positive | Positive | Positive | Negative | 5% | Negative | Benign | RLL Wedge Resection |
| Luo et al^16^ | Female (65) | Cough | | RLL | 2.9 | None | Unk | Positive | Positive | Positive | Positive | 2% | Positive | Benign | RLL Lobectomy w/ LN dissection |
| Hong et al^17^ | Male (54) | Productive cough | | LUL | 2.0 | None | Unk | Positive | Unk | Positive | Unk | No mitosis observed | Unk | Benign | LUL Wedge Resection |
| Zulpaite et al^18^ | Female (43) | None | | LUL | 4.5 | None | Unk | Positive | Positive | Positive | Negative | 2% | Unk | Benign | Wedge resection |
| Huang et al^19^ | Female (44) | Chest pain | | RLL | 2.5 | None | Unk | Positive | Unk | Positive | Positive | 10% | Positive | Benign | Wedge resection |
| Oide et al^20^ | Male (44) | None | | LUL | 2.0 | None | Unk | Positive | Positive | Positive | Unk | Unk | Unk | Benign | "Partial Lung Resection" |
| Juan et al^21^ | Male (55) | None | | LUL | 4.5 | Buccal cancer | Unk | Positive | Positive | Unk | Unk | No mitosis observed | Negative | Benign | Wedge resection |
| Yang et al^22^ | Female (60) | SOB | | LUL | 1.5 | None | 5.2 | Positive | Positive | Unk | Unk | No mitosis observed | Positive | Benign | Wedge Resection |
| Cheng et al.^23^ | Male (57) | None | | RML | 3.2 | None | Unk | Positive | Positive | Positive | Negative | 10% | Unk | Benign | RML wedge resection |
| Feng et al.^24^ | Female (55) | SOB; chest tightness; cough | | LLL | 9.5 | None | 8.1 | Positive | Positive | Unk | Positive | 20% | Positive | Benign | LLL Wedge resection |
| Zhu et al.^25^ | Male (73) | Fatigue | | RLL | 4.1 | None | Unk | Positive | Positive | Negative | Negative | 3% | Negative | Benign | RLL wedge resection |
| Minami et al^26^ | Female (67) | L thigh pain | | LLL -> L Femur | Unk | None | Unk | Positive | Unk | Positive | Unk | 20% | Unk | Malignant | LLL wedge resection; Denosumab and radiation to left thigh |
| Meirelles et al.^27^ | Male (48) | None | | RLL | 1.5 | None | 12.9 | Positive | Unk | Positive | Negative | Unk | Negative | Benign | RLL Lobectomy |
| Cura et al.^28^ | Female (58) | None | | RUL | 2.0 | None | "positive" | Positive | Unk | Positive | Unk | No mitosis observed | Unk | Benign | RUL lobectomy |
| Barrett et al.^29^ | Female (54) | None | | RLL | 1.3 | None | Unk | Positive | Unk | Unk | Unk | Unk | “Positive in adipocytes” | Benign | Segmentectomy with mediastinal LN dissection |
| Baksiyan et al.^30^ | Female (54) | None | | RLL | 1.4 | Cervical cancer | Unk | Positive | Positive | Positive | Unk | Unk | Unk | Benign | RLL Lobectomy |
| Kemnitz et al^31^ | Female (59) | Pain | | RLL | 4.0 | None | Unk | Unk | Unk | Unk | Unk | Unk | Unk | Benign | Wedge resection |
| Huang et al^32^ | Female (64) | None | | RUL, RLL | Unk | None | Unk | Positive | Unk | Positive | Negative | 2% | Negative | Benign | RUL and RLL wedge resections |
| Incarbone et al^33^ | Male (24) | Hemoptysis | | RUL | 2.4 | None | 10.14 | Positive | Unk | Positive | Unk | No mitosis observed | Unk | Benign | RUL wedge Resection |
| Izumi et al^34^ | Female (18) | Hemoptysis | | LUL | 3.0 | None | Unk | Positive | Unk | Positive | Negative | Neg | Positive | Benign | LUL lobectomy |
| Pezzuto et al^35^ | Female (46) | cough and dyspnea | | LUL | 2.6 | None | 4.3 | Positive | Positive | Unk | Unk | No mitosis observed | Unk | Benign | LUL lobectomy with mediastinal and hilar lymphadenectomy |
| Kaneda et al^36^ | Female (49) | None | | LUL | 1.4 | None | Unk | Unk | Unk | Unk | Unk | Unk | Unk | Benign | LUL wedge resection |
| van der Meij^37^ | Female (40) | Dyspnea; wheezing; nocturnal coughing; dysphagia | | RLL | 5.0 | None | Unk | Positive | Positive | Positive | Unk | <5% | Negative | Malignant | right pneumonectomy with mediastinal LN dissection |
| Prayson et al^38^ | Male (51) | None | | RUL | 6.5 | None | Unk | Positive | Positive | Positive | Negative | "Increased Mitosis" | Positive | Malignant | RUL lobectomy |
| Hsu et al^39^ | Male (66) | None | | LLL | 3.0 | None | Unk | Positive | Unk | Unk | Negative | Unk | Unk | Benign | LLL lobectomy |
| Vaideeswar et al^40^ | Female (54) | None | | LUL | 2.0 | None | Unk | Positive | Unk | Positive | Unk | No mitosis observed | Unk | Benign | N/A (found on autopsy) |
| Picquet et al^41^ | Female (54) | None | | LLL | 1.2 | Infiltrating Lobular breast carcinoma | Unk | Positive | Unk | Unk | Unk | No mitosis observed | Unk | Benign | Wedge resection |
| Lepanto et al^42^ | Female (60) | None | | LUL | 1.7 | Infiltrating mixed ductal-lobular breast carcinoma | 1.2 | Positive | Positive | Unk | Unk | Unk | Unk | Benign | Wedge resection |
| Jiang et al^43^ | Female (63) | None | | LUL | 3.5 | None | Unk | Positive | Positive | Positive | Negative | 5%% | Negative | Benign | LUL lobectomy |
| Kim et al^44^ | Female (61) | Chest Pain | | RUL | 2.5 | None | Unk | Unk | Unk | Unk | Unk | Unk | Unk | Benign | RUL lobectomy with mediastinal LN dissection |
| Weber et al^45^ | Female (Unk) | None | | RLL | 15.0 | None | Unk | Positive | Negative | Positive | Negative | 15% | Unk | Benign | N/A (found on autopsy) |
| Rowsell et al^46^ | Male (51) | None | | RLL | 4.0 | None | Unk | Positive | Unk | Positive | Negative | Unk | Negative | Benign | Lobectomy |
| Comin et al^47^ | Male (33) | Hemoptysis; Chest Pain | | LUL | 2.0 | None | Unk | Positive | Positive | Positive | Negative | 5% | Positive | Benign | LUL wedge resection |
| Falleni et al^48^ | Female (45) | None | | RML | 4.5 | None | Unk | Positive | Unk | Positive | Unk | No mitosis observed | Positive | Benign | Right lateral thoracotomy |
| Falleni et al^48^ | Male (59) | None | | LUL | 2.5 | Colonic adenocarcinoma | Unk | Negative | Unk | Positive | Unk | Neg | Positive | Benign | LUL Lobectomy |
| Zhao et al^49^ | Male (59) | None | | RUL | 0.5 | None | Unk | Positive | Unk | Positive | Unk | Unk | Negative | Benign | Wedge resection |
| Ueno et al^50^ | Female (61) | None | | Scattered | 1.5 | None | Unk | Positive | Unk | Positive | Unk | Unk | Unk | Benign | Excised biopsies |
| de Perrot et al^51^ | Female (57) | None | | RLL | 0.9 | Lung adenocarcinoma | Unk | Positive | Unk | Positive | Unk | Unk | Negative | Benign | Wedge Resection |
| Kaleem et al^52^ | Female (45) | None | | Unk | 1.2 | None | Unk | Positive | Unk | Positive | Unk | Unk | Unk | Benign | Unk |
| Lockett et al^53^ | Male (65) | Cough | | LLL | 0.8 | None | Unk | Positive | Unk | Positive | Negative | No mitosis observed | Negative | Benign | wedge resection |
| Maiorana et al^54^ | Male (65) | None | | “Right lung” | 1.8 | None | Unk | Unk | Unk | Unk | Unk | Unk | Unk | Benign | Lobectomy |
| Liu et al^55^ | Male (48) | None | | RLL | 2.1 | None | Unk | Positive | Unk | Negative | Unk | Unk | Positive | Benign | RLL Wedge Resection |
| Drlicek et al^56^ | Male (41) | none | | Unk | 2.5 | None | Unk | Unk | Unk | Unk | Unk | Unk | Unk | Benign | Unk |
| Drlicek et al^56^ | Female (62) | None | | Unk | 6.0 | None | Unk | Unk | Unk | Unk | Unk | Unk | Unk | Benign | Unk |
| Robinson^57^ | Female (55) | None | | RUL | 2.0 | Endometrial adenocarcinoma; ocular melanoma | Unk | Positive | Unk | Positive | Unk | Unk | Negative | Benign | Unk |
| Flynn et al^58^ | Female (63) | Cough; sore throat | | LUL | 3.0 | None | Unk | Positive | Unk | Positive | Unk | No mitosis observed | Negative | Benign | LUL lobectomy |
| Flynn et al^58^ | Female (74) | None | | LLL | 1.7 | None | Unk | Positive | Unk | Positive | Unk | No mitosis observed | Negative | Benign | LLL lobectomy |
| Chumas et al^59^ | Male (58) | None | | Unk | 4.0 | None | Unk | Unk | Unk | Unk | Unk | Unk | Unk | Benign | Unk |
| Zhang et al^60^ | Female (58) | None | | LUL | 2.5 | None | Unk | Unk | Unk | Unk | Unk | Unk | Unk | Benign | LUL lobectomy |
| Kodama et al^61^ | Male (53) | None | | LLL | 2.6 | None | Unk | Unk | Unk | Unk | Unk | Unk | Unk | Benign | Linear segmentectomy |
| Lopez et al^62^ | Female (79) | None | | RLL | Unk | None | Unk | Positive | Positive | Unk | Unk | No mitosis observed | Unk | Benign | RLL wedge resection |
| Ong et al^63^ | Female (69) | None | | RML | 3.8 | None | Unk | Positive | Unk | Positive | Unk | Unk | Positive | Benign | RML lobectomy and mediastinal LN dissection |
| Satoh et al^64^ | Female (64) | None | | Scattered | 2.5 | None | Unk | Positive | Positive | Positive | Unk | Unk | Negative | Benign | Observation |
| Masago et al^65^ | Male (76) | None | | LUL | 1.0 | Poorly differentiated gastric adenocarcinoma | Unk | Positive | Positive | Unk | Unk | 4% | Negative | Benign | Unk |

CD: cluster of differentiation; EMA: epithelial membrane antigen; LLL: left lower lobe; LN: lymph node; LUL: left upper lobe; PET: positron emission tomography; PR: progesterone receptor; RCC: renal cell carcinoma; RLL: right lower lobe; RML: right middle lobe; RUL: right upper lobe; SOX: Sry-related HMG-box; Unk: unknown

1. Zhang DB, Chen T. Primary pulmonary meningioma: A case report and review of the literature. *World J Clin Cases*. 2022;10(13):4196-4206.

2. Dai ZY, Jiang Y, Wang FQ, Wang Y. Primary pulmonary meningioma: A case report and review of the literature. *Asian J Surg*. 2024.

3. Cesario A, Galetta D, Margaritora S, Granone P. Unsuspected primary pulmonary meningioma. *Eur J Cardiothorac Surg*. 2002;21(3):553-555.

4. Yang X, Gao X, Wang S. Primary mediastinal malignant meningioma. *Eur J Cardiothorac Surg*. 2009;36(1):217-218.

5. Oh JH, Cho HS, Hwang HS, Ji W. Primary pulmonary meningioma presenting as multiple lung nodules: A case report. *Thorac Cancer*. 2022;13(1):141-143.

6. Baş A, Valiyev E, Özkan ND, et al. A Rare Entity: Primary Pulmonary Meningioma. *Turk Patoloji Derg*. 2023;39(1):98-99.

7. Jiang M, Chen P, Huang R, Zhang J, Zheng J. A case report of primary pulmonary meningioma masquerading as lung metastasis in a patient with rectal carcinoma: role of. *J Cardiothorac Surg*. 2021;16(1):153.

8. Gürçay N, Öztürk A, Demirağ F, İncekara F. Primary pulmonary meningioma mimicking pulmonary metastasis: A rare case report. *Turk Gogus Kalp Damar Cerrahisi Derg*. 2020;28(4):699-701.

9. Fujikawa R, Arai Y, Otsuki Y, Nakamura T. A case of a primary pulmonary meningioma mimicking a metastasis from a papillary thyroid carcinoma due to a size reduction after radioactive iodine therapy. *Surg Case Rep*. 2020;6(1):57.

10. Wang X, Li P, Zhou P, Fu Y, Lai Y, Che G. Intrapulmonary metastasis from primary pulmonary meningioma presenting as multiple cystic lesions: a case report. *BMC Pulm Med*. 2019;19(1):8.

11. Han D, Deng H, Liu Y. Primary pulmonary meningiomas: report of two cases and review of the literature. *Pathol Res Pract*. 2020;216(12):153232.

12. Cimini A, Ricci F, Pugliese L, Chiaravalloti A, Schillaci O, Floris R. A Patient with a Benign and a Malignant Primary Pulmonary Meningioma: An Evaluation with 18F Fluorodeoxyglucose Positron Emission Tomography/Computed Tomography and Computed Tomography with Iodinated Contrast. *Indian J Nucl Med*. 2019;34(1):45-47.

13. Bae SY, Kim HS, Jang HJ, et al. Primary Pulmonary Chordoid Meningioma. *Korean J Thorac Cardiovasc Surg*. 2018;51(6):410-414.

14. Ohashi-Nakatani K, Shibuki Y, Fujima M, et al. Primary pulmonary meningioma: A rare case report of aspiration cytological features and immunohistochemical assessment. *Diagn Cytopathol*. 2019;47(4):330-333.

15. Xu KK, Tian F, Cui Y. Primary pulmonary meningioma presenting as a micro solid nodule: A rare case report. *Thorac Cancer*. 2018;9(7):874-876.

16. Luo JZ, Zhan C, Ni X, Shi Y, Wang Q. Primary pulmonary meningioma mimicking lung metastatic tumor: a case report. *J Cardiothorac Surg*. 2018;13(1):99.

17. Hong S, Jiang J, Zhou F, Liu J. Computed tomography findings of primary pulmonary meningioma: A case report. *Medicine (Baltimore)*. 2018;97(2):e9651.

18. Žulpaitė R, Jagelavičius Ž, Mickys U, Janilionis R. Primary Pulmonary Meningioma With Rhabdoid Features. *Int J Surg Pathol*. 2019;27(4):457-463.

19. Huang S, Chen L, Mao Y, Tong H. Primary pulmonary meningioma: A case report. *Medicine (Baltimore)*. 2017;96(19):e6474.

20. Oide T, Hiroshima K, Shibuya K, Nakatani Y. Primary Pulmonary Meningioma Presenting as a Coin Lesion. *Intern Med*. 2017;56(15):2073-2074.

21. Juan CM, Chen ML, Ho SY, Huang YC. Primary Pulmonary Meningioma Simulating a Pulmonary Metastasis. *Case Rep Pulmonol*. 2016;2016:8248749.

22. Yang B, Qiu J. Primary pulmonary meningioma with associated multiple micronodules: a case report and literature review. *J Surg Case Rep*. 2023;2023(2):rjad034.

23. Cheng J, Guo C. Primary pulmonary meningioma: a case report and literature review. *J Cardiothorac Surg*. 2025;20(1):44.

24. Feng Y, Wang P, Liu Y, Dai W. PET/CT imaging of giant primary pulmonary meningioma: a case report and literature review. *J Cardiothorac Surg*. 2023;18(1):171.

25. Zhu D, Ou Z, Yan G, Cao J, Xu E. Primary Pulmonary Meningioma With Associated Multiple Micronodules: A Case Report With Comprehensive Diagnostic Overview. *Cancer Rep (Hoboken)*. 2024;7(6):e2123.

26. Minami Y, Sato S, Koyanagi H, Kinowaki Y. Malignant primary pulmonary meningioma with bone metastasis. *Oxf Med Case Reports*. 2020;2020(2):omaa005.

27. Meirelles GS, Ravizzini G, Moreira AL, Akhurst T. Primary pulmonary meningioma manifesting as a solitary pulmonary nodule with a false-positive PET scan. *J Thorac Imaging*. 2006;21(3):225-227.

28. Cura M, Smoak W, Dala R. Pulmonary meningioma: false-positive positron emission tomography for malignant pulmonary nodules. *Clin Nucl Med*. 2002;27(10):701-704.

29. Barrett T, George Z, Khatskevich K, Forcucci JA, Hajar C. Incidental Metaplastic Primary Pulmonary Meningioma. *Int J Surg Pathol*. 2024;32(4):776-779.

30. Baksiyan GA, Zavialov AA, Lishchuk SV. Primary pulmonary meningioma — a rare lung tumor. *Journal of Clinical Practice*. 2024;15(4):110-114.

31. Kemnitz P, Spormann H, Heinrich P. Meningioma of lung: first report with light and electron microscopic findings. *Ultrastruct Pathol*. 1982;3(4):359-365.

32. Huang X, Mou YF, Ren FQ, Wang Y, Yang Y. Multiple primary pulmonary meningioma: A case report and literature review. *Thorac Cancer*. 2022;13(15):2257-2259.

33. Incarbone M, Ceresoli GL, Di Tommaso L, et al. Primary pulmonary meningioma: report of a case and review of the literature. *Lung Cancer*. 2008;62(3):401-407.

34. Izumi N, Nishiyama N, Iwata T, et al. Primary pulmonary meningioma presenting with hemoptysis on exertion. *Ann Thorac Surg*. 2009;88(2):647-648.

35. Pezzuto F, Giraudo C, Baldi M, dell'Amore A, Calabrese F. Primary pulmonary meningioma mimicking a carcinoid tumor in a middle-aged female. *Pathologica*. 2024;116(5):324-327.

36. Kaneda Y, Miyoshi T, Hiratsuka M, et al. [Primary pulmonary meningioma; report of a case]. *Kyobu Geka*. 2005;58(6):512-515.

37. van der Meij JJ, Boomars KA, van den Bosch JM, van Boven WJ, de Bruin PC, Seldenrijk CA. Primary pulmonary malignant meningioma. *Ann Thorac Surg*. 2005;80(4):1523-1525.

38. Prayson R, Farver C. Primary Pulmonary Malignant Meningioma. *The American Journal of Surgical Pathology*;23(6):722-726.

39. Hsu CC, Tsai YM, Yang SF, Hsu JS. Primary pulmonary meningioma. *Kaohsiung J Med Sci*. 2023;39(11):1155-1156.

40. Vaideeswar P, Chaudhari J. Primary pulmonary meningioma. *Indian J Pathol Microbiol*. 2019;62(3):486-487.

41. Picquet J, Valo I, Jousset Y, Enon B. Primary pulmonary meningioma first suspected of being a lung metastasis. *Ann Thorac Surg*. 2005;79(4):1407-1409.

42. Lepanto D, Maffini F, Petrella F, et al. Atypical primary pulmonary meningioma: a report of a case suspected of being a lung metastasis. *Ecancermedicalscience*. 2014;8:414.

43. Jiang G, Zhang Y, Yu J, et al. Primary pulmonary meningioma: a case report and a review of the literature *International Journal of Clinical and Experimental Pathology*. 2016;9(4):4467-4472.

44. Kim YY, Hong YK, Kie JH, Ryu SJ. Primary pulmonary meningioma: an unusual cause of a nodule with strong and homogeneous enhancement. *Clin Imaging*. 2016;40(1):170-173.

45. Weber C, Pautex S, Zulian GB, Pusztaszeri M, Lobrinus JA. Primary pulmonary malignant meningioma with lymph node and liver metastasis in a centenary woman, an autopsy case. *Virchows Arch*. 2013;462(4):481-485.

46. Rowsell C, Sirbovan J, Rosenblum MK, Perez-Ordoñez B. Primary chordoid meningioma of lung. *Virchows Arch*. 2005;446(3):333-337.

47. Comin CE, Caldarella A, Novelli L, Janni A. Primary pulmonary meningioma: report of a case and review of the literature. *Tumori*. 2003;89(1):102-105.

48. Falleni M, Roz E, Dessy E, et al. Primary intrathoracic meningioma: histopathological, immunohistochemical and ultrastructural study of two cases. *Virchows Arch*. 2001;439(2):196-200.

49. Zhao S, Liu X, Xiang M, Dai J. Primary pulmonary meningioma presenting as a pulmonary ground glass nodule: a case report and review of the literature. *J Med Case Rep*. 2024;18(1):350.

50. Ueno M, Fujiyama J, Yamazaki I, Uchiyama T, Ishikawa Y, Satoh Y. Cytology of primary pulmonary meningioma. Report of the first multiple case. *Acta Cytol*. 1998;42(6):1424-1430.

51. de Perrot M, Kurt AM, Robert J, Spiliopoulos A. Primary pulmonary meningioma presenting as lung metastasis. *Scand Cardiovasc J*. 1999;33(2):121-123.

52. Kaleem Z, Fitzpatrick MM, Ritter JH. Primary pulmonary meningioma. Report of a case and review of the literature. *Arch Pathol Lab Med*. 1997;121(6):631-636.

53. Lockett L, Chiang V, Scully N. Primary pulmonary meningioma: report of a case and review of the literature. *Am J Surg Pathol*. 1997;21(4):453-460.

54. Maiorana A, Ficarra G, Fano RA, Spagna G. Primary solitary meningioma of the lung. *Pathologica*. 1996;88(5):457-462.

55. Liu D, Lu X, Shen J, Huang S, Wang H. Primary pulmonary meningioma: A case report. *Turk Gogus Kalp Damar Cerrahisi Derg*. 2023;31(2):286-288.

56. Drlicek M, Grisold W, Lorber J, Hackl H, Wuketich S, Jellinger K. Pulmonary meningioma. Immunohistochemical and ultrastructural features. *Am J Surg Pathol*. 1991;15(5):455-459.

57. Robinson PG. Pulmonary meningioma. Report of a case with electron microscopic and immunohistochemical findings. *Am J Clin Pathol*. 1992;97(6):814-817.

58. Flynn SD, Yousem SA. Pulmonary meningiomas: a report of two cases. *Hum Pathol*. 1991;22(5):469-474.

59. Chumas JC, Lorelle CA. Pulmonary meningioma. A light- and electron-microscopic study. *Am J Surg Pathol*. 1982;6(8):795-801.

60. Zhang FL, Cheng XR, Zhang YS, Ding JA. Lung ectopic meningioma. A case report. *Chin Med J (Engl)*. 1983;96(4):309-311.

61. Kodama K, Doi O, Higashiyama M, Horai T, Tateishi R, Nakagawa H. Primary and metastatic pulmonary meningioma. *Cancer*. 1991;67(5):1412-1417.

62. Lopez J, Miotto A, Botter M, et al. Primary Pulmonary Meningioma in an Elderly Patient: Case Report. *Chest*. 2016;150(4):691A.

63. Ong K, Rajapaksha K, Ahmed A. Primary Pulmonary Meningioma: Rare Tumor with Malignant Potential. *Journal of Thoracic Oncology*. 2017;12(1):S1020.

64. Satoh Y, Ishikawa Y. Multiple primary pulmonary meningiomas: 20-year follow-up findings for a first reported case confirming a benign biological nature. *Int J Surg Case Rep*. 2017;31:58-60.

65. Masago K, Hosada W, Sasaki E, et al. Is primary pulmonary meningioma a giant form of a meningothelial-like nodule? A case report and review of the literature. *Case Rep Oncol*. 2012;5(2):471-478.
